# Valorization of soursop flowers (*Annona muricata* L.) as potent source of natural antioxidants for stabilization of palm olein during accelerated storage

**DOI:** 10.1002/fsn3.349

**Published:** 2016-02-16

**Authors:** Hilaire Macaire Womeni, Fabrice Tonfack Djikeng, Naga Satya Surya Prabhakar Iruku, Mallampalli Sri Lakshmi Karuna, Rachapudi Badari Narayana Prasad, Michel Linder

**Affiliations:** ^1^ Department of BiochemistryFaculty of ScienceUniversity of DschangDschangCameroon; ^2^ CSIR‐Indian Institute of Chemical TechnologyCentre for Lipid ResearchTarnakaHyderabad500007India; ^3^ Biomolecular Engineering Laboratory (LIBio)University of LorraineENSAIAVandoeuvre‐les‐NancyFrance

**Keywords:** Accelerated storage, natural antioxidant, Oxidative stability, palm olein, soursop flowers

## Abstract

This study investigates the effect of soursop flowers methanolic extract on the physicochemical characteristics of palm olein during accelerated storage. After analysis of the extract by determining its total phenolic content by colorimetry and identification of some of its phenolic antioxidants by HPLC‐DAD(Diode Array Detector), preliminary antioxidants tests have been done. After that, the extract was added in palm olein at concentrations 200–1800 ppm. BHT, at 200 ppm served as standard besides the Control. Induction time, peroxide, *p*‐anisidine, Total oxidation (TOTOX), thiobarbituric acid and iodine values, as well as changes in linoleic acid profile Gas Chromatography/Flamme Ionization Detector (GC/FID) of oil during the storage were evaluated. Results showed soursop flower extract to be rich in phenolic antioxidants and to be efficient, at all concentrations, in delaying palm olein oxidation on Rancimat and accelerated Schaal oven test of 30 days at 70°C. Soursop flowers might be used as potent source of antioxidants for the stabilization of palm olein.

## Introduction

Fats, oils, and lipid‐based foods deteriorate through several degradation reactions during heating or long‐term storage. The main deterioration processes is oxidation, which result in the decrement of the nutritional properties of foods, since it involves in the loss of essential fatty acids, essential amino acids, destruction of vitamins, and reduction in protein digestibility (Cuvelier and Maillard 2012). It also reduce the organoleptic properties of foods and may generates potential toxic compounds implicated in human degenerative diseases such as cancer, cardiovascular diseases etc. Due to these changes, consumers do not accept oxidized products and industries suffer from important economic losses Iqbal and Bhanger (2007).

In order to overcome these deterioration reactions, synthetic antioxidants such as butylatedhydroxyanisole (BHA), butylatedhydroxytoluene (BHT), and ter‐butyl hydroquinone (TBHQ) have been used as food additives. However, recent reports reveal their implication in many health risks, including cancer and cardiovascular diseases, and their use is becoming restricted in several countries (Iqbal et al. 2008). In addition, according to Chang et al. (1977) and Thorat et al. (2013), BHA and BHT are quite volatile and easily decomposed at high temperatures. Today, the increasing consumer awareness of synthetic food additives and safety has prompted increase interest in the use of natural antioxidants as alternatives to synthetic antioxidants, because of their potential health benefits (Krishnaiah et al. 2010).

The majority of natural antioxidants are phenolic compounds or polyphenols and their antioxidant activity is based on their structure, hydrogen‐donating potential, ability to chelate metal ions and their synergic actions (Bouba et al. 2010). In many studies, natural plants extracts have been used for stabilization of edible oils (Iqbal et al. 2008; Womeni et al. 2013). But, recently, only few natural sources have been authorized for industrial purpose (case of rosemary) (Cuvelier and Maillard 2012). It is necessary to explore more and more other natural sources.

*Annona muricata* L., family Annonaceae, commonly called ‘Soursop’ is a small, upright evergreen tree growing 5–6 m in height. Its leaves are normally evergreen, 6–20 cm long and 2–6 cm wide. The flowers are borne simply, and may emerge anywhere on the trunk, branches or twigs. They are short stalked, 4–5 cm long, plump, and triangular or conical; the 3 fleshy, slightly spreading, outer petals are yellow/green with 3 closet inner pale‐yellow petals. The fruit is more or less oval or heart‐shaped, sometimes irregular, with the size ranging from 10–30 cm long and up to 15 cm in width. The soursop tree is indigenous to most of the world's tropical rainforest areas of South and North America as well as West African Countries like Nigeria, Ghana, Ivory Coast and Gambia (Olakunle et al. 2014). All parts of the tree are used in natural medicine in the tropics including the stem bark, leaves, root, fruit, and fruit seeds. It has been used as phyto‐therapy for various ailments such as cancer, diabetes hypertension, inflammation broad spectrum internal and external bacterial and fungal infections, helminthes, etc (Olakunle et al. 2014). The plant has also been reported to have good antioxidant property (Baskar et al. 2007). However, only the antioxidant activity of its leaves, fruits and bark have already been reported (Olakunle et al. 2014). No study has been reported on the antioxidant property of its flowers, and their ability to retard oxidation reactions in edible oils and fats. The soursop tree flowers are significantly lost during the flowering because of gusts of wind. The lost flowers could be valorized as potent source of natural antioxidant.

The objective of this study was to evaluate the ability of soursop flowers extract to reduce peroxidation and preserve linoleic acid of palm olein subjected to accelerated oxidation of 30 days at 70°C. Palm olein was used in this study because it is the most produced and consumed refined oil in Cameroon, and also because it is an unsaturated oil.

## Material and Methods

### Material

All chemicals, solvents used in this study were of analytical grade and were procured from Sd Fine Chemicals, HiMedia Laboratories Pvt. Ltd, Mumbai, India and Sigma‐Aldrich, St. Louis. Standard antioxidants and fatty acid methyl esters (C_4_–C_24_) were procured from Sigma‐Aldrich.

Refined, bleached, and deodorized palm olein, free from additives was obtained from SCS/RAFCA Palm Oil Industry Company Ltd, Bafoussam, West Cameroon. Fresh soursop flowers (*Annona muricata*. L) were collected in Dschang Menoua's Department, West Cameroon, on March 2013. All the chemicals and reagents used were of analytical reagent grade.

### Methods

#### Extraction of Soursop flowers antioxidants

Fresh soursop flowers (*Annona muricata*) were dried in an electric air‐dried oven at 50°C for 48 h. The dried flowers were grounded to pass through a 1 mm sieve. About 100 g of the obtained powder was extracted into 800 mL of methanol for 48 h at room temperature. The mixture was regularly subjected to shaking during the extraction. After that, the extract was filtered with a Wattman No. 1 filter paper, and residues again extracted with 400 mL of methanol to ensure the maximum extraction of phenolic compounds. Then, the filtrates were subjected to rotary evaporation at 40°C under reduced pressure for the removal of solvent. The obtained extract was stored at 4°C prior to further analysis.

### Quantitative phytochemical analysis

#### Determination of total phenolics content

Total phenols content was determined using the Folin–Ciocalteu colorimetric method as described by Gao et al. (2000). Plant extracts (20 *μ*L) were mixed in a test tube with 0.2 mL of Folin–Ciocalteu reagent and 2 mL of distilled water and incubated at room temperature for 3 min. Following this, 1 mL of 20% sodium carbonate was added to the mixture, reincubated for 2 h at room temperature. The absorbance of the resulting blue color was measured using a quartz cuvet at 765 nm. Gallic acid was used as standard and total phenolic content was expressed as milligrams equivalents gallic acid per gram of extract.

#### High performance liquid – diode array detection chromatography of the extract

Reverse‐phase HPLC was used to analyze the composition of phenolics in the extract (1 mg/mL in methanol). The HPLC agilent system 1200 series used was equipped with a quaternary pump model G11311A and Diode Array Detector (DAD) model G11315B in combination with Chemstation software. The column type was an RP‐C18 Lichrospher column, 5 *μ*m, 4.0 mm internal diameter × 250 mm. Separations were done in the isocratic mode, using acetonitrile‐1% orthophosphoric acid in water (70:30 v/v) at a flow rate of 1 mL per min; with an injection volume of 20 *μ*L. DAD detection was at 280 nm. Identification of the antioxidants was achieved by comparing their retention time to those of standards. Caffeic acid, vanillic acid, gallic acid, ellagic acid, quercetin, tannic acid, and ferulic acid which are phenolic antioxidants serve as standards.

### Preliminary antioxidant tests

#### DPPH radical scavenging assay

The radical scavenging ability of the extract was determined according to the method of Braca et al. (2002). 4.5 mL of 0.002% alcoholic solution of DPPH was added to 0.5 mL of different concentrations (125, 250, 500, 1000, and 2000 *μ*g/mL) of samples and standard solutions separately, in order to have final concentrations of products of 125–200 *μ*g/mL. The samples were kept at room temperature in the dark and after 30 min and the absorbance of the resulting solution was measured at 517 nm. The absorbance of the samples, control and blank was measured in comparison with methanol. One synthetic antioxidant, butylatedhydroxytoluene (BHT) was used as positive control. The antiradical activity (AA) was determined using the following formula:

AA% = [(Abs_control_ – Abs_sample)_ × 100/Abs_control_]

#### Ferric reducing antioxidant power

The antioxidant potential of soursop flowers extract was also evaluated by their ability to reduce iron (III) to iron (II) following the method of Oyaizu (1986). An aliquot of 0.5 mL plant extract (125, 250, 500, 1000, and 2000 *μ*g/mL) was mixed with 1 mL phosphate buffer (0.2 M, pH 6.6) and 1 mL of 1% aqueous K_3_Fe (CN)_6_ solution, well shaken and incubated at 50°C for 30 min. After incubation, 1 mL of 10% TCA solution was added to stop the reaction and the mixture was centrifuged at 1008 g for 10 min. 1.5 mL of supernatant, 1.5 mL of distilled water and 0.1 mL of 0.1% FeCl_3_ solution were mixed and incubated for 10 min and absorbance read at 700 nm on spectrophotometer. The final concentration of the extract solutions were of 12.5–200 *μ*g/mL. A sample blank, containing all the reagents but no extract was prepared in the same conditions. Catechin, a powerful ferric reducer, was used as positive control to compare the reducing power of the extracts. A higher absorbance indicates a higher reducing power.

### Effect of soursop flowers extract on palm olein oxidation

#### Samples preparation

The crude concentrated methanolic extract of soursop flowers was dissolved in a few volume of methanol and separately added to 100 g of preheated RBD palm olein (at 50°C for 3 h) at concentrations of 200, 600, 1000, 1400, and 1800 ppm. Synthetic antioxidant (Butylated hydroxytoluene) was employed at the legal limit of 200 ppm (Duh and Yen 1997) to compare the efficacy of natural antioxidants. A palm olein sample, free from additives and prepared under the same conditions, was used as control. After the removal of methanol, each sample was separated in two portions, 10 g and 90 g, for Rancimat test and Schaal oven test respectively.

#### Rancimat test

Induction periods of stabilized and control palm olein samples were evaluated using an automated Metrohm Rancimat model 892. About 5 g of each oil sample was weighed in individual reaction vessels of the instrument and vessels were placed in a heating block for 10 min for preheating of sample. After that, air was supplied by a built‐in‐pump at flow rate of 20 L/h. Temperature was adjusted to 110°C and absorption vessels filled with 60 mL deionized water were connected with reaction vessels via teflon tubing. The induction period, the time elapsed from the beginning until the oil starts to become rancid, was automatically recorded, and the protection factors calculated as following: PF = IP_A_/IP_0_ (IP_A_ = induction time of oil with antioxidant and IP_0_ = Induction time of oil without antioxidant) (Yanishlieva‐Maslarova and Heinonen 2001).

#### Schaal oven test

Stabilized and control oil samples (90 g) were placed in dark brown airtight glass bottles with narrow necks and subjected to accelerated storage in an electric hot air oven at 70°C (8 h heating cycle per day) for 30 days. Samples were collected every 10 days and stored in the refrigerator for further analysis. The oxidative deterioration level was assessed by the measurement of peroxide, p‐anisidine, Total oxidation (TOTOX), thiobarbituric acid, iodine values.

#### Measurement of oxidation parameters

Determination of the peroxide value of stabilized and control palm olein samples were made following the spectrophotometrical IDF standard method, 74A: 1991 (International Dairy Federation 1991). p‐Anisidine, iodine, and free fatty acid values assays was carried out according to the procedure in AOCS Official Method CD 18–90 (American Oil Chemists’ Society 1999). Total oxidation (TOTOX) values of oil samples were determined using the equation TOTOX = 2PV + AV according to Shahidi and Wanasundara (2008). Thiobarbituric acid values were evaluated according to Draper and Hadley (1990).

### Effects of soursop flowers extract on the linoleic acid profile of palm olein during the storage

#### Preparation of fatty acids methyl esters

Fatty acid methyl esters (FAMEs) of stabilized and control palm olein samples were prepared by transesterification using 2% sulfuric acid in methanol (Christie 1993). The FAMEs were extracted into ethyl acetate and thoroughly washed with water to make them free of acid and dried over anhydrous sodium sulfate. The dried esters were analyzed in GC/FID. All the analyses were conducted in duplicate.

#### Gas chromatography

The GC‐FID analyses were performed with an Agilent (Agilent Technologies, Palo Alto, CA) 7890A series gas chromatograph equipped with an FID detector using a DB‐225 capillary column (30 m × 0.25 mm, 0.25 *μ*m of film thickness). The column temperature was initially maintained at 160°C for 2 min, increased to 220°C at 5°C per min and maintained for 10 min at 220°C. The carrier gas was nitrogen at a flow rate of 1.5 mL per min. The injector and detector temperatures were maintained at 230 and 250°C, respectively, with a split ratio of 50:1. Identification of linoleic acid was based on comparison of GC retention time with that of standard reference fatty acid methyl esters performed under the same conditions.

### Statistical analysis

The tests were performed at least in duplicate and results are representative of the mean ± standard deviations. Results have been submitted to the statistical analysis of variance (ANOVA) at 0.05% probability level. The Dunnett and Student–Newmann–Keuls tests were used to compare means using the software Graphpad‐InStat (Flamme Ionization Detector).

## Results and Discussion

### Extraction yield, total phenolic content, and phenolic antioxidants detected by HPLC

The extraction yield of soursop flowers methanolic extracts was found to be 8.35%. This value was less than that obtained by George et al. (2012) with butanolic extracts of soursop leaves (10.80%). Concerning the total phenolic content of this extract, it was found to be 51.33 mg/g. This value was higher than that found by George et al. (2012)in butanolic extracts of soursop leaves. In fact, these authors have evaluated the TPC of different quantities of soursop leaves butanolic extract: 25, 50, 100, 200, and 400 *μ*g and have found TPC values to be 6.60, 7.37, 8.31, 11.54, and 37.42 *μ*g, respectively. From these observations, it seems that soursop flowers contain a higher amount of phenolic compounds than leaves. Environmental differences, part of plant tested, time of samples collection, and determination methods could explain the observed variations.

**Figure 1:**
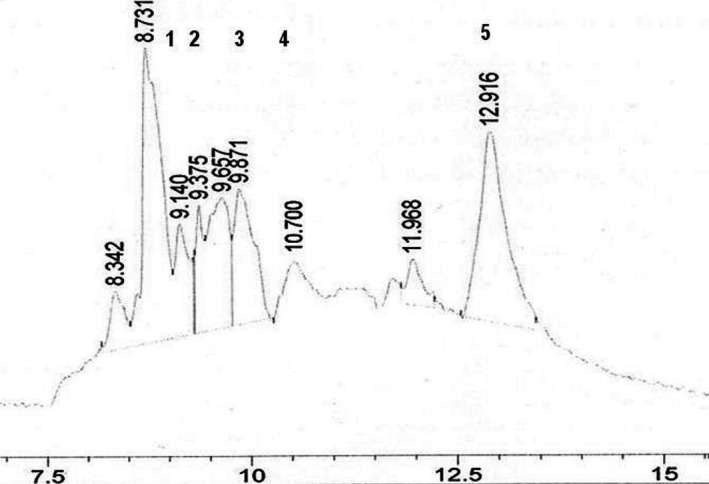
HPLC‐DAD(Diode Array Detector) Chromatogram of soursop flowers methanolic extract. Peak 1 = vanillic acid, peak 2 = caffeic acid, peak 3 = ferulic acid, peak 4 = ellagic acid and peak 5 = quercetine.

The HPLC‐DAD analysis of soursop flowers methanolic extract is shown in Figure 1. The peaks 1, 2, 3, 4, and 5 were found as matching well with vanillic acid (RT: 9.140 min; peak area: 7.74%), caffeic acid (RT: 9.375 min; peak area: 4.90%), ferulic acid (RT: 9.871 min. peak area: 13.07%), ellagic acid (RT: 10.700. peak area: 3.54%), and quercetin (12.916 min; peak area: 25.34%), respectively. Aside from that, there were a few unidentified peaks, which needed further analysis. It is well known that the detected phenolic compounds in this extract are strong antioxidants. Quercetin was the most abundant compound among the antioxidants detected. It presence in this extract is in accordance with the findings of Georges et al. (2014) who have detected the same compound in the alcoholic extract of soursop leaves.

### Preliminary antioxidant tests

#### DPPH and FRAP power tests

**Figure 2:**
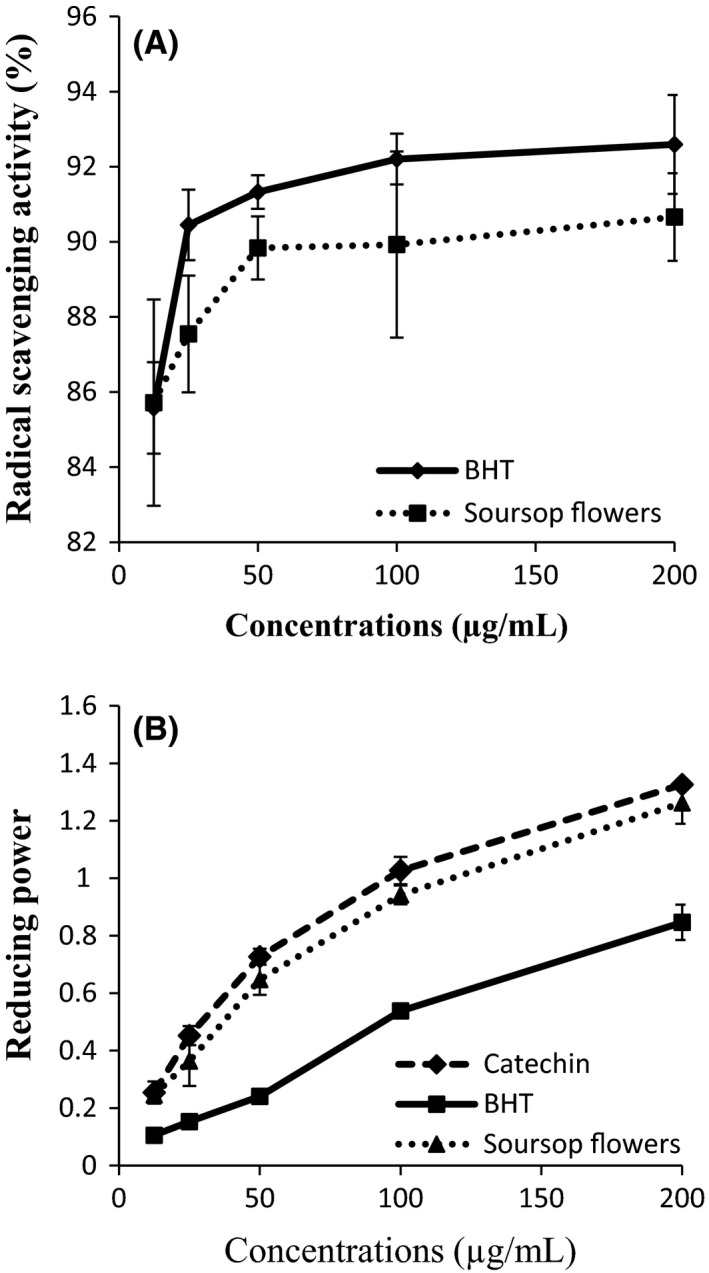
(A–B) DPPH radical scavenging activity (A) and Ferric reducing antioxidant power (B) of Soursop flowers extract (*Annona muricata*. L).

DPPH and FRAP (Ferric Reducing Antioxidant Power) tests were used to assess the free radical scavenging activity and electron‐donating capacity of the extract, and results are illustrated in Figure 2. Both assays showed that the antioxidant activity of the extract significantly increase with the concentration. The radical scavenging activity (Fig. 2A) of the extract was similar (*P *>* *0.05), at all concentration, to that of BHT. The same observations were made between the extract and Catechin in their reducing power (Fig. 2B). These results testify the powerful antioxidant property of soursop flowers. BHT was shown to be a powerful radical scavenger, but a weak metal reducer. Similar results, showing that plants flowers can be a powerful radical scavenger and ferric reducers have been previously reported by Barreira et al. (2008) with Chestnut flowers. The observed activity could be attributed to the presence of phenolic antioxidants suspected in this extract. In fact, in many studies, the antioxidant activity of plant extract is attributed to these compounds, and it is believed that the number of hydroxyl group present their aromatic constituents as well as their position offer better antioxidative properties (Chen and Ho 1997; Roginsky and Lissi 2005). These results are in accordance with those of Baskar et al. (2007) who showed that soursop leaves ethanolic extract is a powerful antioxidant.

### Effects of Soursop flowers extract on palm olein oxidative stability

#### Rancimat test

This test has been performed in order to evaluate the efficiency of the extract in delaying palm olein oxidation and results (induction time and protection factors) are presented in Table 1. It is clearly observed that induction time and protection factors of oil samples containing antioxidants were significantly higher (*P *<* *0.05) than that of control. At all concentrations, the activity of the extract was significantly elevated (*P *<* *0.05) than that of BHT, showing it good thermal stability than BHT and good antioxidant activity. Induction time and protection factors of oil samples supplemented with the extract were increasing with the concentration of antioxidants. This result is agreement with that of Iqbal and Bhanger (2007) and Iqbal et al. (2008) who showed that, the induction time of sunflower oil supplemented with garlic and pomegranate methanolic extract, respectively, was higher than that of the control and was increasing with the concentration of extract. The fact that PO + BHT has a lowest induction period value and protection factor than samples containing our natural plant extract might be attributed to it instability at elevated temperature. Chang et al. (1977) and Thorat et al. (2013) showed that BHT and BHA are quite volatile and easily decompose at high temperatures. The observed efficiency of the extract in prolonging induction time of palm olein as well as its good protection factors might be attributed to the phenolic antioxidants present in this extract.

**Table 1:** Induction time, protection factor of oil samples and their Changes in peroxide, *p*‐anisidine, and Total oxidation (TOTOX) values during storage

Characteristic	Day	Control	PO + BHT_200 ppm_	PO + An.M_200 ppm_	PO + An.M_600 ppm_	PO + An.M_1000 ppm_	PO + An.M_1400 ppm_	PO + An.M_1800 ppm_
Induction time (h)	–	20.08 ± 0.14^a^	22.66 ± 0.36^b^	24.07 ± 0.16^c^	24.29 ± 0.89^c^	26.18 ± 0.33^d^	27.28 ± 0.26^de^	28.38 ± 0.68^e^
Protection factor	–	1.00 ± 0.00^a^	1.13 ± 0.00^b^	1.19 ± 0.00^c^	1.21 ± 0.03^c^	1.30 ± 0.00^d^	1.36 ± 0.00^e^	1.41 ± 0.04^e^
Peroxide value (ppm)	0	8.88 ± 1.23^a^_A_	8.88 ± 1.23^a^_A_	8.88 ± 1.23^a^_A_	8.88 ± 1.23^a^_A_	8.88 ± 1.23^a^_A_	8.88 ± 1.23^a^_A_	8.88 ± 1.23^a^_A_
	10	27.70 ± 0.26^a^_B_	13.78 ± 0.00^b^_B_	21.74 ± 0.52^c^_B_	13.73 ± 0.32^b^_B_	12.57 ± 0.98 ^d^_B_	12.17 ± 1.40^d^_B_	10.42 ± 0.15^e^_B_
	20	27.90 ± 1.13^a^_B_	18.82 ± 0.32^b^_C_	24.86 ± 0.27^c^_C_	21.11 ± 0.70^b^_C_	17.31 ± 0.58^b^_C_	14.09 ± 0.10^d^_B_	12.98 ± 0.01^d^_C_
	30	30.31 ± 0.17^a^_C_	23.25 ± 0.64^b^_D_	23.59 ± 2.16^b^_BC_	23.85 ± 1.83^b^_D_	22.38 ± 0.00^b^_D_	21.83 ± 0.69^b^_C_	19.70 ± 0.13^c^_D_
*p*‐Anisidine value	0	7.82 ± 0.77^a^_A_	7.82 ± 0.77^a^_A_	7.82 ± 0.77^a^_A_	7.82 ± 0.77^a^_A_	7.82 ± 0.77^a^_A_	7.82 ± 0.77^a^_A_	7.82 ± 0.77^a^_A_
	10	12.04 ± 0.93^a^_B_	7.80 ± 0.24^b^_A_	8.80 ± 0.46^d^_A_	7.02 ± 0.21^c^_BA_	7.83 ± 0.23^b^_A_	7.11 ± 0.00^c^_BA_	7.07 ± 0.00^c^_B_
	20	12.04 ± 0.00^a^_B_	8.36 ± 0.72^b^_A_	9.33 ± 1.10^b^_AB_	8.16 ± 1.48^b^_A_	8.38 ± 0.94^b^_A_	7.40 ± 0.61^b^_A_	7.23 ± 1.37^b^_A_
	30	14.61 ± 0.00^a^_C_	11.81 ± 0.06^b^_B_	10.55 ± 0.00^c^_B_	9.71 ± 0.53^c^_CA_	8.58 ± 0.00^d^_A_	8.43 ± 0.00^d^_CA_	7.67 ± 0.61^e^_A_
TOTOX VALUE	0	25.58 ± 1.31^a^_A_	25.58 ± 1.31^a^_A_	25.58 ± 1.31^a^_A_	25.58 ± 1.31^a^_A_	25.58 ± 1.31^a^_A_	25.58 ± 1.31^a^_A_	25.58 ± 1.31^a^_A_
	10	67.20 ± 1.47^a^_B_	34.80 ± 0.73^b^_B_	52.28 ± 1.52^c^_B_	34.49 ± 0.86^b^_B_	32.97 ± 2.20^bc^_B_	31.47 ± 0.30^bc^_B_	27.92 ± 2.81^c^_A_
	20	69.67 ± 2.27^a^_B_	46.01 ± 0.90^b^_C_	59.06 ± 1.64^c^_C_	50.39 ± 2.88^d^_C_	43.01 ± 2.11^b^_C_	35.60 ± 0.81^e^_C_	33.21 ± 1.40^e^_B_
	30	75.25 ± 0.35^a^_C_	58.33 ± 1.34^b^_D_	64.80 ± 2.33^b^_C_	62.03 ± 4.21^b^_D_	59.52 ± 0.00^b^_D_	54.58 ± 1.38^c^_D_	41.59 ± 1.88^d^_C_

(a–e) Means within each row with different superscripts are significantly (*P *<* *0.05) different. (A–D) Means within each column with different superscripts are significantly (*P *<* *0.05) different. (Control: Palm olein without antioxidant; PO + BHT 200 ppm: palm olein containing BHT as antioxidant at concentration of 200 ppm; PO + An.M_200_: palm olein supplemented with the extract at concentration of 200 ppm).

#### Schaal oven test

##### Peroxide value

Table 2 shows the relative increase in peroxide value of palm olein treatments under accelerated storage. The peroxide value, which measures hydroperoxides products of the oil, is a good indicator of the oxidation state of the oil. A typical pattern in the rise of peroxide value was observed for almost all the oil samples. The control had exhibited the highest peroxide value among all the treatments showing a higher degree of oxidation. In the oil samples containing soursop flowers as source of natural antioxidants, PO + AnM_1800 ppm_, PO + AnM_1400 ppm,_ PO + An.M _1000 ppm_, and PO + An.M_600 ppm_ were found to be the most resistant against peroxides formation. The peroxide formation in these samples was similar or less than that of PO + BHT_200 ppm_ proof of the efficiency of the extract in retarding primary oxidation products formation in palm olein. PO + An.M_200 ppm_ had exhibited a highest peroxide value than the other stabilized oils, but, the formation of peroxides in this sample was significantly (*P* < 0.01) lower compared to the control. Results clearly indicate a good antioxidant and concentration‐dependent activity of our extract, as obtained on Rancimat. This might be due to the effect of the phenolic antioxidants present. Similar efficacy and concentration‐dependent activity have been obtained by Iqbal and Bhanger (2007) and Iqbal et al. (2008) with methanolic extract of garlic and pomegranate, respectively, in sunflower oil. These results are in agreement with those found in the literature (Navas et al. 2006; Iqbal et al. 2008; Mei et al. 2014).

**Table 2:** Changes in TBA, iodine values, and linoleic acid content of RBD palm olein during storage

Characteristic	Day	Control	PO + BHT_200 ppm_	PO + An.M_200 ppm_	PO + An.M_600 ppm_	PO + An.M_1000 ppm_	PO + An.M_1400 ppm_	PO + An.M_1800 ppm_
TBA value (ppm)	0	0.85 ± 0.00^a^_A_	0.85 ± 0.00^a^_A_	0.85 ± 0.00^a^_A_	0.85 ± 0.00^a^_A_	0.85 ± 0.00^a^_A_	0.85 ± 0.00^a^_A_	0.85 ± 0.00^a^_A_
	10	1.94 ± 0.01^a^_B_	0.93 ± 0.09^bc^_AB_	1.79 ± 0.03^c^_B_	1.39 ± 0.14^b^_B_	1.10 ± 0.08^b^_B_	1.05 ± 0.05^b^_B_	0.93 ± 0.00^c^_B_
	20	2.00 ± 0.00^a^_B_	1.00 ± 0.17^bd^_AB_	1.81 ± 0.03^c^_B_	1.70 ± 0.07^c^_C_	1.28 ± 0.12^b^_B_	1.01 ± 0.18^bd^_AB_	0.90 ± 0.06^d^_AB_
	30	2.22 ± 0.06^a^_C_	1.09 ± 0.02^b^_B_	2.18 ± 0.08^a^_C_	1.84 ± 0.03^c^_C_	1.34 ± 0.04^d^_C_	1.13 ± 0.10^b^_B_	1.12 ± 0.03^b^_C_
Iodine value (g I_2_/100 g)	0	57.73 ± 0.04^a^_A_	57.73 ± 0.04^a^_A_	57.73 ± 0.04^a^_A_	57.73 ± 0.04^a^_A_	57.73 ± 0.04^a^_A_	57.73 ± 0.04^a^_A_	57.73 ± 0.04^a^_A_
	10	56.90 ± 0.00^a^_B_	57.46 ± 0.10^b^_B_	56.91 ± 0.10^a^_B_	57.14 ± 0.01^c^_B_	57.12 ± 0.00^c^_B_	57.18 ± 0.02^c^_B_	57.32 ± 0.09^b^_B_
	20	55.86 ± 0.03^a^_C_	57.09 ± 0.05^b^_C_	56.71 ± 0.04^c^_A_	57.06 ± 0.01^b^_C_	56.86 ± 0.09^b^_C_	57.07 ± 0.01^b^_C_	57.12 ± 0.07^b^_C_
	30	55.16 ± 0.07^a^_D_	56.38 ± 0.05^b^_B_	56.46 ± 0.05^b^_C_	56.54 ± 0.11^bc^_C_	56.63 ± 0.07^c^_D_	56.90 ± 0.06^d^_D_	57.03 ± 0.19^cd^_C_
Linoleic acid profile (%)	0	10.97 ± 0.03^a^_A_	10.97 ± 0.03^a^_A_	10.97 ± 0.03^a^_A_	10.97 ± 0.03^a^_A_	10.97 ± 0.03^a^_A_	10.97 ± 0.03^a^_A_	10.97 ± 0.03^a^_A_
	10	10.43 ± 0.01^a^_B_	10.86 ± 0.06^b^_A_	10.45 ± 0.05^a^_B_	10.58 ± 0.04^c^_B_	10.62 ± 0.05^c^_B_	10.58 ± 0.07^c^_B_	10.64 ± 0.04^c^_B_
	20	10.09 ± 0.00^a^_C_	10.50 ± 0.03^b^_B_	10.11 ± 0.05^a^_C_	10.49 ± 0.02^b^_B_	10.37 ± 0.08^c^_C_	10.59 ± 0.04^b^_B_	10.72 ± 0.03 ^d^_B_
	30	10.02 ± 0.05^a^_C_	9.22 ± 0.06^b^_C_	10.27 ± 0.03^c^_D_	10.16 ± 0.08^c^_C_	10.40 ± 0.05^e^_C_	10.45 ± 0.03^e^_C_	10.57 ± 0.12^f^_B_

(a–f) Means within each row with different superscripts are significantly (*P *<* *0.05) different. (A–D) Means within each column with different superscripts are significantly (*P *<* *0.05) different. (Control: Palm olein without antioxidant; PO + BHT 200 ppm: palm olein containing BHT as antioxidant at concentration of 200 ppm; PO + An.M_200_: palm olein supplemented with the extract at concentration of 200 ppm).

##### *p*‐anisidine value

Table 1 also illustrate the level of formation of secondary oxidation products (expressed as *p*‐anisidine value) in stabilized and control palm olein samples. The measurement of this parameter is intensively used to assess secondary oxidation products, mainly nonvolatile carbonyls (2‐alkenal and 2, 4‐alkadienal) formed during the lipid oxidative degradation (Anwar et al. 2010). The periodical analysis of oil samples revealed a significant (*P *<* *0.001) increase in *p*‐anisidine value of control compared to oil samples containing natural or synthetic antioxidants. The *p*‐anisidine value of oils containing the extract was not far from that enriched with BHT during the storage period, confirming the efficiency of soursop extract in inhibiting the formation of secondary oxidation products in palm olein, by blocking hydroperoxides decomposition. This might be due to the variable mechanisms of action of phenolic compounds present in this extract. The obtained results, showing that soursop flowers extracts significantly prevent secondary oxidation products formation in palm olein during an accelerated storage, are in accordance with those found by others authors in different oils and with different natural plants extract (Anwar et al.*,*).

##### TOTOX value

Totox value measures both hydroperoxides and their breakdown products, and provides a better estimation of the progressive oxidative deterioration of fats and oils. In Table 1 is illustrated the changes in TOTOX value of stabilized and Control palm olein samples during the storage. The highest degree of oxidation was obtained with palm olein without antioxidant (Control) while palm olein samples supplemented with natural or synthetic antioxidants were very less oxidized. The resistance toward oxidation in samples containing the natural extract was concentration‐dependent and increased as follows: PO + An.M_200 ppm_< PO + An.M_600 ppm_< PO + AnM_1000 ppm_< PO + AnM_1400 ppm_< PO + An.M_1800 ppm_. However, their TOTOX values were significantly lower (*P* < 0.001) than that of the Control, but not far from that of PO + BHT_200 ppm_. This is the proof of the good antioxidant property of our extracts in stabilizing palm olein. This result confirms the concentration‐dependent effect of our natural antioxidant obtained by the Rancimat method, and the good correlation between Rancimat and Schaal oven test. The effect of natural plant extract in limiting total oxidation of vegetable oils has already been proven (Mei et al. 2014).

##### Thiobarbituric acid value

The TBA test is an easy and quick method widely used for the assessment of secondary product of oxidation in which malonaldehyde is quantified (Iqbal and Bhanger 2007). Changes in thiobarbituric acid value of palm olein samples supplemented with different concentration of soursop flowers extract in comparison with Control and PO + BHT_200 ppm_ are illustrated in Table 2. The control has exhibited the highest TBA value, followed by PO + An.M_200 ppm_. At the 30th day, the TBA value of PO + An.M_200 ppm_ was not different (*P *>* *0.05) from that of the control, whereas, at days 10 and 20, that value became significantly lower (*P *<* *0.05) than that of control. However, the TBA value of oil samples concentrated at 600–1800 ppm with the extracts have presented lowest (*P *<* *0.001) values compared to the Control. As previously observed with the Rancimat, peroxides and TOTOX values, the activity of the extracts was concentration‐dependent. BHT, An.M_1000_, An.M_1400_, and AnM_1800 ppm_ were most efficient in retarding formation of malonaldehyde in palm olein. From these results, it can be concluded that soursop flowers methanolic extract can inhibit malonaldehyde formation in palm olein. This activity could be attributed to the good antioxidant potential of phenolic compounds present in this extract. Similar results have been obtained by Mei et al. (2014) and Iqbal et al. (2008) in sunflower oil using Rambutan and pomegranate extracts, respectively.

##### Iodine value

Palm olein contains more than 50% unsaturated fatty acids. These unsaturated fatty acids are susceptible to lipid oxidation. During storage, the double bonds of these unsaturated fatty acids are attacked by free radicals, which results in the formation of conjugated bonds (Tynek et al. 2001). Thus, measuring the amount of unsaturated fatty acids present in this oil can be used as a reference to determine the freshness of the oil. The iodine values of the stabilized and control palm olein samples, over an incubation period of 30 days at 70°C, are shown in Table 2. It is observed that the iodine value decreases gradually during the storage. This decrement, as previously mentioned is generally attributed to the destruction of the fatty acid double bonds caused by oxidation process (Tynek et al. 2001). The rate of decrement in Control was significantly higher (*P *<* *0.05) than that of stabilized oils, showing that the added antioxidants have significantly reduced the alteration of fatty acids double bonds in palm olein. The activity of the extract was not far from that of BHT. From these results it can be concluded that soursop flowers methanolic extract, at all concentrations, could effectively delay the oxidation of unsaturated fatty acids in palm olein. Its protective effect is comparable to that of BHT. Antioxidants compounds present in this extract could be responsible of the observed activity. Similar results have been reported by Mei et al. (2014) in sunflower oil and Womeni et al. (2013) in soybean oil with extract of Rambutan (*Nephelium lappaceum* L) and some Cameroonian spices, respectively, during accelerated storage of 24 days at 65°C.

### Effect of soursop flowers extract on the linoleic acid profile of palm olein during the storage

The effect of soursop flowers on the linoleic acid profile of palm olein during the storage is presented in Table 2. At the start, the linoleic acid percent of not heated palm olein was 10.97%. After 30 days heating, these values significantly decreased until 10.02, 9.22, 10.27, 10.16, 10.40, 10.45, and 10.57%, respectively, for PO, PO + BHT_200 ppm_, PO + An.M_200 ppm_, PO + An.M_600 ppm_, PO + An.M_1000 ppm_, PO + An.M_1400 ppm_, and PO + An.M_1800 ppm._ As previously mentioned with the iodine value, this decrement might be the consequence of the rapid oxidation of linoleic acid acids in favor to primary and secondary oxidation products. It appears clear that the linoleic acid percent of oil containing the extract was significantly higher than that of the control and PO + BHT_200 ppm_ at the 30th storage day, showing that linoleic acid was most preserved by our natural extract from the oxidative degradation. Similar results have been reported by Che man and Tan ((1999) in palm olein and Navas et al. (2006) in sunflower oil.

## Conclusion

From this study, it is clear that soursop flowers contain a good amount of phenolic compound and is a potent free radical scavenger and ferric reducer. Its activity in palm olein, tested by the Rancimat method was better than that of BHT due to its potential good thermal stability at high temperatures and synergic action of its antioxidants. However, its activity was similar to that of BHT when evaluated by the Schaal oven test. Soursop flowers can be exploited as an alternative source of antioxidants for the stabilization of palm olein and other oil systems.

## Conflict of Interest

The authors declare that they have no conflict of interest.
